# Perceptions of Dutch health care professionals regarding the Care Standard for diabetes

**DOI:** 10.1186/1756-0500-6-417

**Published:** 2013-10-17

**Authors:** Lieke GM Raaijmakers, Marloes K Martens, Charlotte Bagchus, Nanne K de Vries, Stef PJ Kremers

**Affiliations:** 1 Department of Health Promotion, NUTRIM School for Nutrition, Toxicology and Metabolism, Maastricht University Medical Centre+, Maastricht, The Netherlands; 2 ResCon, research & consultancy, Haarlem, The Netherlands; 3 Athena Institute, VU University, Amsterdam, The Netherlands; 4 Caphri, School for Primary Care and Public Health, Maastricht University Medical Centre+, Maastricht, The Netherlands

**Keywords:** Diabetes, Care Standard, Health care professionals, Barriers

## Abstract

**Background:**

The Netherlands can be regarded as unique in the use of the Netherlands Diabetes Federation (NDF) Care Standard (CS) for diabetes. The need to understand the barriers obstructing optimal health care, the dissemination and implementation of health care innovations into daily practice and the extent to which health care professionals actually adhere to guidelines has been emphasized repeatedly. Therefore, the aim of the present study was to suggest ways to optimize the implementation of the CS by examining the perceptions of Dutch health care professionals regarding the CS and the barriers to using it.

**Methods:**

A cross-sectional questionnaire survey was conducted among health care professionals (N = 1547) in 2010.

**Results:**

A total of 39.6% (N = 1323) of the participating health care professionals possessed the CS. Only 15.5% of the professionals who were to some extent familiar with the CS (N = 1100) described themselves as working in complete accordance with the CS. The majority (83.9%) thought the CS contributed greatly to ensuring the quality of care; the judgment on the feasibility of working in accordance with the CS was positive (mean = 3.9 on a 5-point Likert scale). However, professionals tended to perceive the guidelines issued by the own professional association as the norm for high quality diabetes care, rather than the CS. The main barrier to using the CS was the lack of effective lifestyle interventions (or access to them) to provide care for people with diabetes or those at increased risk for the disorder.

**Conclusions:**

A limited percentage of health care professionals were found to posses the CS. It is questionable whether possession of the CS is a prerequisite for delivering high quality care. Overall, professionals were largely positive about the CS, although only a minority indicated they were working in complete accordance with it. Professionals and professional organizations should be further educated about the content of the CS and especially its added value with respect to the guidelines for their own professional group, in terms of the multidisciplinary approach to diabetes care. Furthermore, attention should be given to the most important perceived barriers, to facilitate adherence to the CS.

## Background

Diabetes mellitus is a rapidly growing health problem, which affects approximately 371 million people worldwide [1]. The prevalence of diabetes in the Netherlands is approaching 1 million (6% of the total population) and this number is increasing by 87,000 patients each year [2]. Diabetes is a complex chronic illness, since it affects various organs and systems and is often accompanied by other diseases. Hence, diabetes requires continuous medical care and ongoing patient self-management and support to prevent acute complications and reduce the risk of complications in the long run [3]. Continuity of care is concerned with the quality of health care for patients with chronic conditions like diabetes and can be achieved when services are seamlessly linked and this is facilitated by shared management plans or care protocols [4,5].

In the Netherlands, attention to continuity of care has increased as a result of the initiative of the Dutch Ministry of Health, Welfare and Sport to start an integrated, programmatic approach of chronic diseases [6]. The concept of continuity of care is also reflected in the Chronic Care Model (CCM), a framework that can be used to optimize the provision of care to patients with chronic conditions [7], and that advocates integrated care and disease management and the use of evidence-based care standards and guidelines [8]. The CCM focuses on improving and optimizing six key elements of the health care system: community resources and policies, organization of health care, self-management support, delivery system design, decision support and clinical information systems [7]. In Dutch diabetes care, the CCM is reflected in the National Diabetes Action Program (NAD), which is funded by the Ministry of Health, Welfare and Sport [9]. The overall purpose of the National Diabetes Action Program (2009–2013) is to create the circumstances, conditions and instruments necessary to slow down the increase in the number of people with diabetes and to reduce complications in diabetes patients [9]. The main objective of the action program is the systematic implementation of the Netherlands Diabetes Federation (NDF) Care Standard (CS) for the content, organization, quality and funding of diabetes prevention and care [9]. A Care Standard is a general framework outlining the services and treatment of people with a specific condition on a very aggregated level, while clinical guidelines describe the content of care in more detail, including how, when and by whom care should be provided [10,11]. The NDF CS for type 2 diabetes mellitus describes the norm for generic multidisciplinary diabetes care and focuses on the content, organization and quality of diabetes care. The CS is constantly being updated and extended, and is based on evidence-based guidelines [12]. The CS functions as a general overarching framework for the guidelines for the individual professional groups and focuses on a multidisciplinary approach to diabetes care. In addition, the CS is used as a purchasing instrument within the Dutch bundled payment approach. In the Netherlands, insurers purchase the services and care as described in the CS from a general contractor (called the Care Group), which ends up in a so called bundled payment contract. Based on this contract, the Care Group assumes financial and clinical accountability and in turn subcontracts individual care providers (like the GP, dietician, internal specialist, etc.) or delivers parts of the services itself.

The Netherlands can be regarded as unique in the use of the CS for diabetes [10]. The first Dutch CS for diabetes was published in April 2003 at the request of, and with financial support by the Ministry of Health, Welfare and Sport, in collaboration with the ZN healthcare insurers association [11,12]. The NDF has adopted a leading role in the implementation of the CS in the Netherlands. In the NDF’s view, it is necessary to use up-to-date, evidence-based guidelines with multidisciplinary support in the care process, to prevent diabetes, to detect it at an early stage and to provide proper treatment [13]. The introduction of the first CS in 2003 coincided with the development of the 'bundled payment’ approach for integrated chronic care [14]. This approach has laid the foundation for delivering and funding diabetes care in accordance with the CS. A project to update the CS was started in 2007, and was announced by the NDF through professional journals and conferences and through their contacts with the NDF members. In 2009, the NDF started the NAD, whose main objective was the systematic nationwide implementation of the Care Standard [9].

In the Netherlands, monodisciplinary guidelines for diabetes, such as the clinical practice guidelines issued by the Dutch College of General Practitioners (NHG), have long been available and have been implemented successfully by general practitioners. The need to understand the barriers obstructing optimal health care, the dissemination and implementation of health care innovations into daily practice and the extent to which health care professionals actually adhere to guidelines has been emphasized repeatedly [15,16]. Previous studies have identified several of these barriers, operating at different levels in the health care system (i.e. the level of the patient, the individual professional, the health care team, the health care organization or the wider environment) [15,17], but not specifically for the use of the CS in the Netherlands. With regard to characteristics of individual professionals, results from a review showed that the main barrier to implementing clinical guidelines is professionals’ limited familiarity with or the lack of awareness of particular guidelines [18,19]. In addition, attitudinal barriers among clinicians and their lack of knowledge about recent evidence-based guidelines have been suggested to impede faithful and complete implementation [20,21]. A study of clinicians’ attitudes showed, however, that guidelines are seen as helpful resources for advice and as valuable educational tools that might improve quality [20]. Studies comparing perceptions of guidelines and standards between different groups of health care professionals have been scarce, and the evidence seems inconsistent [22]. There are, however, some indications that general practitioners are more reluctant to follow guidelines than professionals working in hospitals [22]. Moreover, a recent study in Norway found significant differences between general practitioners and other medical doctors in terms of their attitudes toward clinical guidelines [22]. As regards environmental factors, limited time, staff shortages and pressure of work have been found to be the main barriers to guideline implementation [18,19].

The aim of the present study was to suggest ways to optimize the implementation of the CS by examining the perceptions of Dutch health care professionals regarding the CS and the barriers to using it. We decided to specifically assess the problem from the perspective of the health care professionals, since this provides us with insights into the perceptions of all involved disciplines that have to work according to the same principles. The specific research questions addressed in this study were: (1) To what extent are health care professionals familiar with the CS and do they perceive themselves as working in accordance with the CS? (2) How do health care professionals appreciate the CS? (3) Are their differences in adherence to and the appreciation of the CS between subgroups (in terms of age, gender, profession and health care division)? (4) Which barriers do health care professionals perceive in working in accordance with the CS?

## Methods

### Design and procedure

A cross-sectional questionnaire survey was conducted among health care professionals (N = 1547) between June and November 2010. The most important strategy used to recruit participants, involved a mailing of personalized letters with a link to a web-based questionnaire for all professions involved in diabetes care: general practitioners (N = 443), practice nurses (N = 441), diabetes nurses (N = 224), dieticians (N = 163), physiotherapists (N = 142), internal medicine physicians (N = 68) and pediatricians (N = 66). The addresses were acquired either from the databases of the professional associations of the relevant professions (e.g. the Dutch Dietetic Association) or by purchasing randomly selected commercially available addresses of health care providers’. In addition, an invitation to participate was placed on the websites and/or in the newsletter of several professional associations of health care providers (e.g. the Dutch College of General Practitioners). Ethical approval was not required for this study.

### Measures

*General characteristics* that were assessed included gender, age and the health care division the professional was working in (i.e. primary or secondary care).

*Familiarity with the CS* was measured with one item asking participants whether they were familiar with the CS. Answer options were: I possess the CS; I have seen it, but do not possess it; I have heard about it, but do not possess it; and I am unfamiliar with it. This variable was recorded into a dichotomous 'possession of CS’ variable ('does not possess’ (1) – 'possesses it’ (2)). *Professionals’ perception of their own adherence to the CS* was assessed with one item, asking participants to what extent they perceived themselves as working in accordance with the CS (1 = not at all; to 5 = completely). This variable was recorded into a dichotomous variable ('working not at all or only partly in accordance with the CS’ (1) – 'working in complete accordance with the CS’ (2)). *Appreciation of the CS* was measured with three items. Two items, scored on 5-point Likert scales (1 = not at all; to 5 = completely), assessed whether professionals regarded the CS as the norm for high quality diabetes care and whether they regarded the guidelines issued by the own professional association as the norm for high quality diabetes care. A third item asked whether they thought the CS contributed to ensuring quality of care ('no contribution’ (1) – 'major contribution’ (3)). This variable was recorded into a dichotomous 'quality assurance’ variable ('no or only minor contribution’ (1) – 'major contribution’ (2)) and analyzed separately. The *feasibility of working in accordance with the CS* was assessed with one item asking whether participants thought it was feasible to work in accordance with the CS (1 = not at all; to 5 = completely). *Barriers* were measured by asking participants to indicate, on a list of items, which one(s) they experienced as barriers. Examples of barriers included 'lack of effective lifestyle interventions (or access to them) to provide care for people with diabetes or at increased risk of diabetes’, 'financial, legislative and regulations issues impeding care and prevention in accordance with the CS’ and 'collaboration between primary and secondary care’. Multiple answers were allowed and an open answer category was provided to add further perceived barriers.

The questionnaire was developed in close collaboration with advisors of the Dutch Diabetes Federation and health promotion research experts and pretested among at least two professionals of each professional group involved in our study.

### Statistical analyses

Data were analyzed using SPSS 17.0. Descriptive statistics and frequencies were used to analyze the respondents’ familiarity with and appreciation of the CS. Associates of working in accordance with the CS and CS quality assurance were determined using logistic regression analyses, via the ENTER method, with age, gender, profession and health care division as independent variables. Similar linear regression analyses were conducted to determine associates of appreciation of the CS and the feasibility of working in accordance with the CS. Contrasts between associates of the independent variables mentioned above were tested by repeating all regression analyses using a different reference group for each categorical variable each time. P-values <0.05 were considered to be statistically significant.

## Results

The mean age of the professionals was 46.9 (SD 8.6) years and the majority were female (66.3%). Of the professionals, 77.3% were working in primary care and 22.7% were working in secondary care.

### Familiarity with and adherence to the Care Standard

Of the health care professionals who answered the question on familiarity with the CS for diabetes (N = 1323), 39.6% possessed the standard, while 19.7% had seen the CS, but did not possess it; 23.8% had heard about it, but did not possess it and 16.9% were unfamiliar with it. This last group was excluded from further analyses. Table 1 shows the familiarity with the CS for each of the professional groups. Diabetes nurses were most likely to possess the CS, followed by dieticians and internal medicine physicians, whilst physiotherapists were the least likely to possess the CS. Of the health care professionals who were to some extent familiar with the CS (N = 1100), 15.5% (which is 11.0% of all respondents) perceived themselves as working in complete accordance with the CS.

**Table 1:** Possession of the Care Standard by professional group

**Health care professionals**	**N**	**Familiarity with Care Standard (%)**	
		***In possession***	***Not in possession***
General practitioner	431	40.4	59.6
Practice nurse	376	35.4	64.6
Diabetes nurse	187	55.1	44.9
Dietician	124	48.4	51.5
Physiotherapist	79	15.2	84.8
Internal medicine physician	65	41.5	58.5
Pediatrician	61	24.6	75.4

### Appreciation of the Care Standard

Table 2 presents the appreciation of the CS among the professionals who were familiar with it. The respondents tended to perceive the guidelines issued by the own professional association as the norm for high quality diabetes care, rather than the CS. Diabetes nurses and dieticians were the least positive about the CS being the norm for high quality diabetes care, even though a relatively large part of the former group (55.4%) possessed the CS. Nevertheless, the majority of the respondents thought the CS made a major contribution to ensuring the quality of care. Working in accordance with the CS was mostly judged to be feasible.

**Table 2:** Associates of heath care professionals’ appreciation of and adherence to the Care Standard (N = 1081)

	**Working in accordance with CS (%)**	**CS as norm (mean(SD))**	**Guidelines for professional group as norm (mean(SD))**	**Feasibility (mean(SD))**	**Quality assurance (%)**
	***Completely***				***Major contribution***
**All participants**	15.5	2.9 (1.0)	4.0 (0.4)	3.9 (0.7)	83.9
**Age**	OR = 1.03*	β = -0.02	β = -0.36	β = 0.05	OR = 1.00 (0.98-1.02)
**Gender**					
Men (1)	18.0	3.3 (1.0)	4.0 (0.3)	4.0 (0.7)	83.0
Women (2)	14.1	2.7 (1.0)	4.0 (0.5)	3.9 (0.7)	84.3
Significant contrasts		2 < 1			
**Professional group**					
General practitioners (1)	27.8	3.0 (1.0)	4.0 (0.2)	4.1 (0.7)	82.1
Practice nurses (2)	12.7	2.7 (0.7)	4.0 (0.3)	3.9 (0.5)	90.0
Diabetes nurses (3)	15.1	2.3 (0.9)	4.1 (0.4)	3.8 (0.7)	89.7
Dieticians (4)	3.4	2.1 (0.7)	4.0 (0.7)	3.6 (0.7)	78.6
Physiotherapists (5)	1.4	2.7 (0.9)	3.8 (0.9)	3.5 (0.9)	75.7
Internal medicine physicians (6)	7.8	3.2 (1.2)	3.8 (0.8)	3.7 (0.9)	64.0
Pediatricians (7)	19.6	3.4 (1.1)	4.0 (0.0)	3.7 (0.9)	73.9
Significant contrasts	2 < 1,7	2,3,4,5 < 1,6,7;	5 < 1,2,3,4,7	2,3,4,5,6,7 < 1	6 < 1,4
	4,5 < 1,2,3,7	3,4 < 2,5	6 < 3	4,5 < 2,3	1,4,5,6,7 < 2,3
	5 < 6				5 < 1
**Health care division**					
Primary care (1)	17.2	3.0 (1.0)	4.0 (0.4)	4.0 (0.7)	85.4
Secondary care (2)	9.3	2.5 (1.1)	4.0 (0.6)	3.7 (0.8)	78.9
Significant contrasts	2 < 1	2 < 1			

*p < 0.05, Note: significant contrasts were identified by comparisons made between the subgroups by repeating the logistic or linear regression analysis using a different reference group for each independent variable each time.

### Associates of adherence to and appreciation of the CS

Table 2 also provides an overview of differences between subgroups in terms of working in accordance with the CS and appreciation of the CS. It shows that the younger respondents were less likely to perceive themselves as working in complete accordance with the CS. Female respondents were less positive about the CS being the norm for high quality diabetes care. Respondents working in secondary care were less likely to perceive themselves to be working in complete accordance with the CS and were less positive about the CS being the norm than respondents working in primary care. Dieticians and physiotherapists were less likely to work in complete accordance with the CS than general practitioners, practice nurses, diabetes nurses, internal medicine physicians and pediatricians. Practice nurses, diabetes nurses, dieticians and physiotherapists were less positive about the CS being the norm than general practitioners, internal medicine physicians and pediatricians. By contrast, physiotherapists were less positive about the guidelines issued by the own professional being the norm than general practitioners, practice nurses, diabetes nurses, dieticians and pediatricians. Compared to general practitioners, all other participating professional groups gave a lower score for the feasibility of using the CS. Finally, practice nurses and diabetes nurses were significantly more positive about the contribution of the CS to ensuring quality of care than the other professional groups.

### Perceived barriers

A total of 68.4% of the respondents reported perceiving barriers to diabetes care; the mean number of reported barriers was 3.1 (SD 2.1, range 1–16). Table 3 presents the three most frequently mentioned perceived barriers, both in general and for each participating professional group separately. In general, the most important barrier was the lack of effective lifestyle interventions (or access to them) to provide care for people with diabetes (or at increased risk for diabetes); this was also the dominant barrier reported by the general practitioners, practice nurses and internal medicine physicians. The diabetes nurses and dieticians perceived the care for groups who are difficult to reach, such as ethnic minorities and people with a low SEP, as the most important barrier. The physiotherapists reported the financial, legislative and regulations issues as the most important barrier to providing care and prevention in accordance with the CS. Pediatricians reported the lack of refresher courses about counseling for self-management as the most important barrier. All respondents, except the internal medicine physicians, listed the financial, legislative and regulations issues among their top five barriers.

**Table 3:** Top 3 barriers perceived, by professional group (N = 687)

**Health care professionals**	**Perceived barriers**	**%**
All participants	Lack of effective lifestyle interventions (or access to them) to provide care for people with diabetes (or at increased risk for it)	41.0
	Care for groups that are difficult to reach, such as ethnic minorities and people with a low SEP	36.3
	Financial, legislative and regulations issues regarding care and prevention in accordance with the Care Standard	31.1
General practitioners	Lack of effective lifestyle interventions (or access to them) to provide care for people with diabetes (or at increased risk for it)	55.4
	Care for groups that are difficult to reach, such as ethnic minorities and people with a low SEP	27.5
	Lack of refresher courses about lifestyle counseling	24.6
Practice nurses	Lack of effective lifestyle interventions (or access to them to provide care for people with diabetes (or at increased risk for it)	39.6
	Financial, legislative and regulations issues regarding care and prevention in accordance with the Care Standard	30.0
	Care for groups that are difficult to reach, such as ethnic minorities and people with a low SEP	28.8
Diabetes nurses	Care for groups that are difficult to reach, such as ethnic minorities and people with a low SEP	59.3
	Financial, legislative and regulations issues regarding care and prevention in accordance with the Care Standard	43.1
	Lack of effective lifestyle interventions (or access to them) to provide care for people with diabetes (or at increased risk for it)	35.4
Dieticians	Care for groups that are difficult to reach, such as ethnic minorities and people with a low SEP	45.7
	Financial, legislative and regulation issues regarding care and prevention in accordance with the Care Standard	43.2
	Care for specific groups, such as pregnant women and people with a depression	34.4
Physiotherapists	Financial, legislative and regulations issues regarding care and prevention in accordance with the Care Standard	42.9
	Care for specific groups, such as pregnant women and people with a depression	38.9
	Care for groups that are difficult to reach, such as ethnic minorities and people with a low SEP	38.8
Internal medicine physcians	Lack of effective lifestyle interventions (or access to them) to provide care for people with diabetes (or at increased risk for it)	55.0
	Standardized recording and exchange of information	36.6
	Care for groups that are difficult to reach, such as ethnic minorities and people with a low SEP	35.0
Pediatricians	Lack of refresher courses about counseling in self-management	37.5
	Financial, legislative and regulations issues regarding care and prevention in accordance with the Care Standard	28.2
	Care for groups that are difficult to reach, such as ethnic minorities and people with a low SEP	25.6

Note: multiple answers allowed.

## Discussion

The main aim of this study was to examine the perceptions of health care professionals regarding the Care Standard for type 2 diabetes of the Netherlands Diabetes Federation (NDF) and the barriers to its implementation. Three years after the introduction of the updated CS in 2007, only one third of the health care professionals actually possessed the CS, while 17% were totally unfamiliar with it. Previous studies found limited familiarity with clinical guidelines to be a major barrier for their implementation [18,19]. It is, however, questionable whether possession of the CS is a prerequisite for delivering high quality care. In the Netherlands, continuity of care has been introduced as part of an integrated, programmatic approach to chronic diseases, which means that professionals working in primary care are subcontracted by a larger Care Group, which is most often exclusively owned by GPs [11,14]. These Care Groups often appear to develop their own care standards and guidelines, but these are typically based on the CS and other evidence-based guidelines. Professionals do not always seem to be aware of this approach, which results in a low reported familiarity with the CS, whereas the care is still delivered according to the CS in routine practice [23]. Involving these Care Groups in future studies assessing perceptions regarding the Care Standard, similar to studies on the Bundled Payment system in the Netherlands [24], would be very useful.

Almost two-thirds of the professionals in our survey who were to some extent familiar with the CS thought the CS contributed greatly to ensuring the quality of care, and the feasibility of working in accordance with the CS was largely endorsed. These results are in line with the findings of studies of clinicians’ attitudes towards guidelines, which showed that clinicians agreed that guidelines do contribute to the quality of care [20]. At present, however, Dutch health care professionals tend to perceive the guidelines issued by the own professional association as the norm for high quality diabetes care, more so than the CS. Recent research has suggested a lack of knowledge about or acceptance of more recent guidelines to be a reason for the lack of adherence to guidelines [25]. Only a small number of the health care professionals in our study who were to some extent familiar with the CS perceived themselves as working in complete accordance with it (15.3%).

The majority of our respondents perceived barriers in implementing diabetes care or in relation to working in accordance with the CS. Overall, we found a lack of effective lifestyle interventions (or access to them) to be the most important perceived barrier, although programs meeting requirements for cost-effective lifestyle interventions have been developed in the Netherlands and have been implemented in several pilot projects in primary care, examples being 'Exercise therapy’ [26] and 'Exercise on prescription’ [27]. The familiarity with such programs, as well as access to them, should obviously be improved. A registration and assessment system for health education and health promotion interventions has been developed in the Netherlands in an attempt to promote quality assurance and control [28]. Whether this system will help ensure that the most effective and efficient interventions are implemented and disseminated can as yet not be guaranteed [29], but at least a comprehensive national list is being put together. Another important perceived barrier we identified was that of financial, legislative and regulations-related issues regarding care and prevention in accordance with the CS. A recent study in the Netherlands examining the fine-tuning of care for chronic conditions also showed that facilities for prevention and self-management were not frequently reimbursed by health insurance companies because these facilities had not yet been finalized [29]. Systematic consultations with relevant stakeholders (such as the Health Insurance Board, the Dutch Healthcare Authority and the Ministry of Health, Welfare and Sport) are needed to ensure that all elements of the CS are covered by health insurance.

We identified several differences between subgroups of our respondents in terms of working in accordance with the CS and their appreciation of the CS. Younger professionals were less likely to perceive themselves as working in complete accordance with the CS. In contrast, results from three reviews showed that young or less experienced professionals were more inclined to use guidelines than older, experienced professionals [19]. The general practitioners in our study were most likely to perceive themselves as working in accordance with the CS, much more so than internal medicine physicians. Diabetes nurses were less positive about CS being the norm for high quality diabetes care than general practitioners and internal medicine physicians. As regards the feasibility of working in accordance with the CS, the general practitioners were more positive than diabetes nurses and internal medicine physicians. Internal medicine physicians were the least positive about the guidelines issued by the own professional association as the norm for high quality diabetes care and the contribution of the CS to ensuring quality of care. In contrast to these findings, a study among Norwegian professionals found general practitioners to demonstrate greater uncertainty and confusion in relation to their views on the volume, legal status, accessibility, evidence base and clarity of clinical guidelines in general, compared to other medical doctors [22]. An explanation for these contrasting findings may be that unlike clinical guidelines, the CS focuses on a multidisciplinary approach in which the general practitioner is considered to assume the role of a director of diabetes care (with the opportunity to delegate some care tasks to the practices nurses and diabetes nurses). Further external validation of our results is limited by the lack of studies comparing perceptions of guidelines between different groups of health care professionals, and by the inconsistent evidence [22].

A strength of the current study is that we included a large sample of health care professionals that consisted of a broad range of professions involved in diabetes care. Since the application of the Care Standard for diabetes is unique in the Netherlands, the results of this study can inform similar future approaches in other countries.

Some limitations need to be acknowledged. Since our study sample was self-selected, it is plausible that the current sample had greater affinity and involvement in diabetes care compared to the entire population. Nevertheless, the representativeness of the sample of health care professionals is indicated by the percentage of general practitioners working in a care group (77.1%), which is in line with the estimated national percentage (78.0%) [30]. Moreover, all professions were represented in our sample in proportion to their total numbers in the Netherlands. Another limitation is that the results are based on self-reported data, which may have led to bias (e.g. through factors related to social desirability). A review comparing studies of adherence to guidelines using observational data and self-reported data showed that self-reported adherence frequently exceeded the observed adherence [19]. Finally, a limited number of factors were assessed, in order to limit the burden to the respondents.

## Conclusions

Familiarity with the Netherlands Diabetes Federation Care Standard (CS) for type 2 diabetes among health care professionals appears to be limited. Despite the respondents’ positive judgment about the feasibility of working in accordance with the CS and about the contribution of the CS to quality assurance, they tended to perceive the guidelines issued by the own professional association as the norm for high quality diabetes care, rather than the CS. The National Diabetes Action Program would therefore be well advised to further educate health care professionals and professional organizations about the content of the CS and especially its added value relative to the guidelines for their own professional groups, in terms of the multidisciplinary approach to diabetes care. Furthermore, attention should be paid to the main perceived barriers to working in accordance with the CS. Therefore, professionals should be educated about the existence of effective lifestyle interventions and their access to these programs should be improved. In addition professionals need to be supported in providing care for groups that are difficult to reach and the financial, legislative and regulations issues relating to care and prevention in accordance with the CS.

## Abbreviations

CCM: Chronic care model; NAD: National diabetes action program; NDF: Netherlands diabetes federation; CS: Care standard; CG: Care group.

## Competing interests

All authors declare that they have no competing interests.

## Authors’ contributions

LR drafted the manuscript. All authors made substantial contributions to its conception and design. LR, CB, MM en SK made substantial contributions to the data acquisition. LR has conducted the analyses and all authors made substantial contributions to the interpretation of the data and have read and approved the final manuscript.
